# Association of static balance parameters with prediabetes: a cross-sectional study and nomogram development

**DOI:** 10.3389/fendo.2026.1921238

**Published:** 2026-08-26

**Authors:** Zhishan Wang, Liangliang Huang, Chen Lin, Jingran Liang, Yuanya Xie, Miaoyun Li, Xingyu Li, Lingjing Gao, Dehong Pan, Jingyu Wang, Luyi Chen, Changbin You, Jiaxiang Liu, Xiaoling Wang, Shujiao Chen

**Affiliations:** 1 Department of Preventive Treatment of Disease, The Third Affiliated People’s Hospital of Fujian University of Traditional Chinese Medicine, Fuzhou, China; 2 Clinical Research Center for Traditional Chinese Medicine (TCM) Clinical Medicine in Diabetes and Lipid Disorders of Fujian Province, Fuzhou, China; 3 College of Traditional Chinese Medicine, Fujian University of Traditional Chinese Medicine, Fuzhou, China; 4 Emergency Department, The Third Affiliated People’s Hospital of Fujian University of Traditional Chinese Medicine, Fuzhou, China; 5 College of Rehabilitation Medicine, Fujian University of Traditional Chinese Medicine, Fuzhou, China; 6 College of Acupuncture and Tuina, Fujian University of Traditional Chinese Medicine, Fuzhou, China; 7 Department of Preventive Treatment of Disease, Affiliated People’s Hospital of Fujian University of Traditional Chinese Medicine, Fuzhou, China

**Keywords:** prediabetes, static balance function, associated factors, candidate functional indicators, nomogram

## Abstract

**Objective:**

To investigate the association between static balance function parameters and prediabetes, and to develop a nomogram integrating center-of-pressure path length, Romberg ratios, and sex, thereby evaluating its potential as a candidate functional screening tool.

**Methods:**

A cross-sectional observational design was adopted. A total of 97 patients with prediabetes and 44 age-matched healthy controls were consecutively enrolled from January 2024 to June 2025 at the Third People’s Hospital Affiliated to Fujian University of Traditional Chinese Medicine. General characteristics and static balance parameters under four sensory conditions were compared between groups. Variables were first screened via Least Absolute Shrinkage and Selection Operator (LASSO) regression, followed by multivariable logistic regression to identify independent associations. A nomogram was then constructed. Model discrimination and calibration were assessed using the receiver operating characteristic curve, Hosmer–Lemeshow test, and 1000-bootstrap internal validation (corrected C-statistic, calibration slope/intercept, Brier score). Effect sizes were quantified using Cohen’s d.

**Results:**

Compared with the healthy control group, the prediabetes group exhibited significantly higher values for Y-COP and COP-PL under Condition 1, RRA and RRT under Condition 2/1, as well as Y-COP, COP-EA, and COP-PL under Conditions 3 and 4 (all *P* < 0.05). LASSO regression retained T1 COP-PL, T3 COP-PL, T4/T3 RRA, and RRT as candidate variables. Multivariable logistic regression showed that female sex (vs. male, OR = 0.208, 95% CI: 0.044–0.973; P = 0.046), T1 COP-PL (per unit increase, OR = 0.990, 95% CI: 0.980–0.999), T3 COP-PL (per unit increase, OR = 1.016, 95% CI: 1.008–1.023), and T4/T3 RRA (per unit increase, OR = 1.013, 95% CI: 1.007–1.020) were independently associated with prediabetes. A nomogram incorporating these variables yielded an AUC of 0.972. After bootstrap internal validation, the corrected AUC was 0.964 (95% CI: 0.941–0.991), with a calibration slope of 0.806, intercept of –0.011, and Brier score of 0.075, indicating good discrimination and calibration. Cohen’s dvalues for T1 COP-PL, T3 COP-PL, and T4/T3 RRA were –0.40, 1.45, and 1.39, respectively, reflecting moderate to large effect sizes.

**Conclusions:**

Static balance indices including T1 COP-PL, T3 COP-PL, and T4/T3 RRA are associated with prediabetes. The nomogram combining these measures with sex demonstrated robust performance in internal validation, offering a functional perspective that may aid in the auxiliary screening of prediabetes. Nevertheless, as a cross-sectional exploratory study, these associations do not imply causality, and the model requires external validation before its clinical generalizability can be determined.

## 1 Introduction

Prediabetes (Pre-DM) is defined as an intermediate hyperglycemic state bridging normoglycemia and diabetes mellitus (DM). It represents a major risk factor for the development of DM, with annual progression rates from Pre-DM to diabetes estimated at 5%-10%. This condition is also associated with the early onset of diabetic complications (1). According to the 11th edition of the IDF Diabetes Atlas (https://diabetesatlas. org/), approximately 589 million adults aged 20–79 years worldwide were living with diabetes, while 635 million had impaired glucose tolerance (IGT) and 488 million had impaired fasting glucose (IFG). Individuals with Pre-DM face significantly elevated risks, including a 13% increase in all-cause mortality, a 15% rise in cardiovascular disease events, a 28% higher risk of diabetic retinopathy, and a 32% greater risk of nephropathy compared to those with normoglycemia (2). DM has thus emerged as a fastest-growing public health challenge of the 21st century. The neurological damage, somatosensory impairment, visual decline, and vestibular injury associated with Type 2 diabetes (T2DM) are key pathophysiological mechanisms underlying balance abnormalities, thereby promoting the development of diabetic sensory complications (3, 4). This progression is characterized by a concomitant decline in balance ability and sensory function as the disease advances.

The study revealed that patients with T2DM exhibited significant impairment in maintaining static postural stability and static balance function. This deficit was markedly exacerbated under conditions of eye closure or sensory perturbation, indicating a heavy reliance on visual compensation to maintain an upright stance (5, 6). The observed balance disorders are primarily associated with diabetic complications, including peripheral neuropathy, visual impairment, and vestibular dysfunction (7–9), which collectively lead to a decline in the central nervous system’s capacity to integrate proprioceptive, visual, and vestibular inputs. As a critical juncture in the pathogenesis of DM, Pre-DM warrants investigation into potential early subclinical complications, including balance disorders. Nevertheless, studies specifically addressing static balance function in Pre-DM are scarce. Our preliminary investigations have shed some light on this issue, demonstrating inverse correlations of 2-hour postprandial glucose (2hPG) and hemoglobin A1c (HbA1c) with vestibular function and proprioception, and of HOMA-IR and FIns with vestibular function and visual input (10). This work lays the groundwork for elucidating the pathophysiological mechanisms linking glucose metabolism abnormalities to incipient static balance dysfunction in Pre-DM.

In this cross-sectional investigation, probed the links between static postural control metrics and Pre-DM. Center-of-pressure path length and Romberg ratios were quantified via a balance feedback platform under four sensory conditions, capturing subtle functional shifts early in the prediabetic continuum. Least Absolute Shrinkage and Selection Operator (LASSO) -regularized logistic regression identified variables independently associated with Pre-DM status, from which a nomogram was constructed to render the strength of these associations visually interpretable. Model performance was rigorously evaluated through receiver operating characteristic analysis, the Hosmer-Lemeshow test, and 1,000-bootstrap internal validation—yielding an optimism-corrected C-statistic, calibration slope and intercept, and the Brier score—while effect sizes were expressed as Cohen’s d. By framing static balance parameters as candidate functional indicators rather than conventional biomarkers, this work adds an observational dimension that complements existing biochemical diagnostic frameworks for Pre-DM.

## 2 Materials and methods

### 2.1 Study design and participant recruitment

This observational study was conducted at the Third People’s Hospital Affiliated to Fujian University of Traditional Chinese Medicine. A total of 141 participants were enrolled and divided into two groups (1) Healthy Control Group (HCG):44 individuals with no history of diabetes; (2) Pre-DM Group:97 adults diagnosed with Pre-DM according to the criteria outlined in the Chinese Guidelines for the Prevention and Control of Type 2 Diabetes (2020 edition) (11) :IFG: 6.1 mmol/L≤Fasting Blood Glucose (FBG)<7.0mmol/L, with 2hPG<7.8 mmol/L;IGT (Impaired Glucose Tolerance):FBG<7.0 mmol/L, with7.8mmol/L≤2hPG<11.1mmol/L.; (3) Written informed consent was obtained from all study participants, including those with prediabetes and healthy controls.

Inclusion Criteria: (1) The inclusion criteria for the Pre-DM group were as follows: 1) aged 18 years or above; 2) meeting the diagnostic criteria for Impaired Fasting Glucose (IFG) and/or Impaired Glucose Tolerance (IGT) defined by the aforementioned guidelines; 3) voluntar participation and provision o written informed consent. (2) Selection of contemporaneous health controls: ① Aged ≥18 years and undergoing routine health examinations during the same period as the prediabetes cohort; ② Oral glucose tolerance test (OGTT) results meeting the following criteria: fasting plasma glucose <6. 1 mmol/L, 2-hour postload glucose <7. 8 mmol/L, and HbA1c <5. 7%; ③ Absence of prior diagnosis of prediabetes or diabetes mellitus, and no current use of glucose-lowering agents; ④ No clinically evident conditions known to impair static balance function; ⑤ No severe dysfunction of major organs, including cardiac, hepatic, or renal systems; ⑥ Provision of written informed consent prior to enrollment.

Exclusion Criteria: (1) Subjects with missing medical records, inability to cooperate with the study procedures, or voluntary withdrawal; (2) Individuals diagnosed with endocrine diseases other than Pre-DM; (3) Cases where balance dysfunction was attributed to exogenous factors such as medication side effects or toxin exposure; (4) Patients with disorders of the hip, knee, or ankle joints, limb amputations, or severe musculoskeletal conditions (e. g., severe kyphosis, significant scoliosis) that could compromise postural stability; (5) Individuals suffering from severe psychiatric disorders (e. g. , major depressive disorder, mania) or significant cognitive impairment that would prevent completion of the assessments; (6) Patients with central nervous system diseases (e. g. , Parkinson’s disease) or disorders related to the vestibular/auditory nerves (e. g. , vestibular neuritis, acoustic neuroma); (7) Subjects with visual/visual field impairments or perceptual abnormalities such as spatial neglect that could affect the evaluation of balance function.

The study was reviewed and approved by the Institutional Review Board(Medical Ethics Committee of The Third People’s Hospital Affiliated to Fujian University of Traditional Chinese Medicine (Approval No. 2023KS-85-1)) and complied with the principles of the Declaration of Helsinki. Written informed consent was obtained from all participants.

### 2.2 Data acquisition

#### 2.2.1 Static balance function was assessed​

Static stability testing was conducted using a stabilometric platform (Pro-Kin PK-254, TecnoBody, Italy) operating in the static assessment module. Prior to testing, the clinician calibrated the device to alternate between eyes-open (EO) and eyes-closed (EC) protocols and provided standardized verbal instructions regarding the procedure and required cooperation.

**Figure 1:**
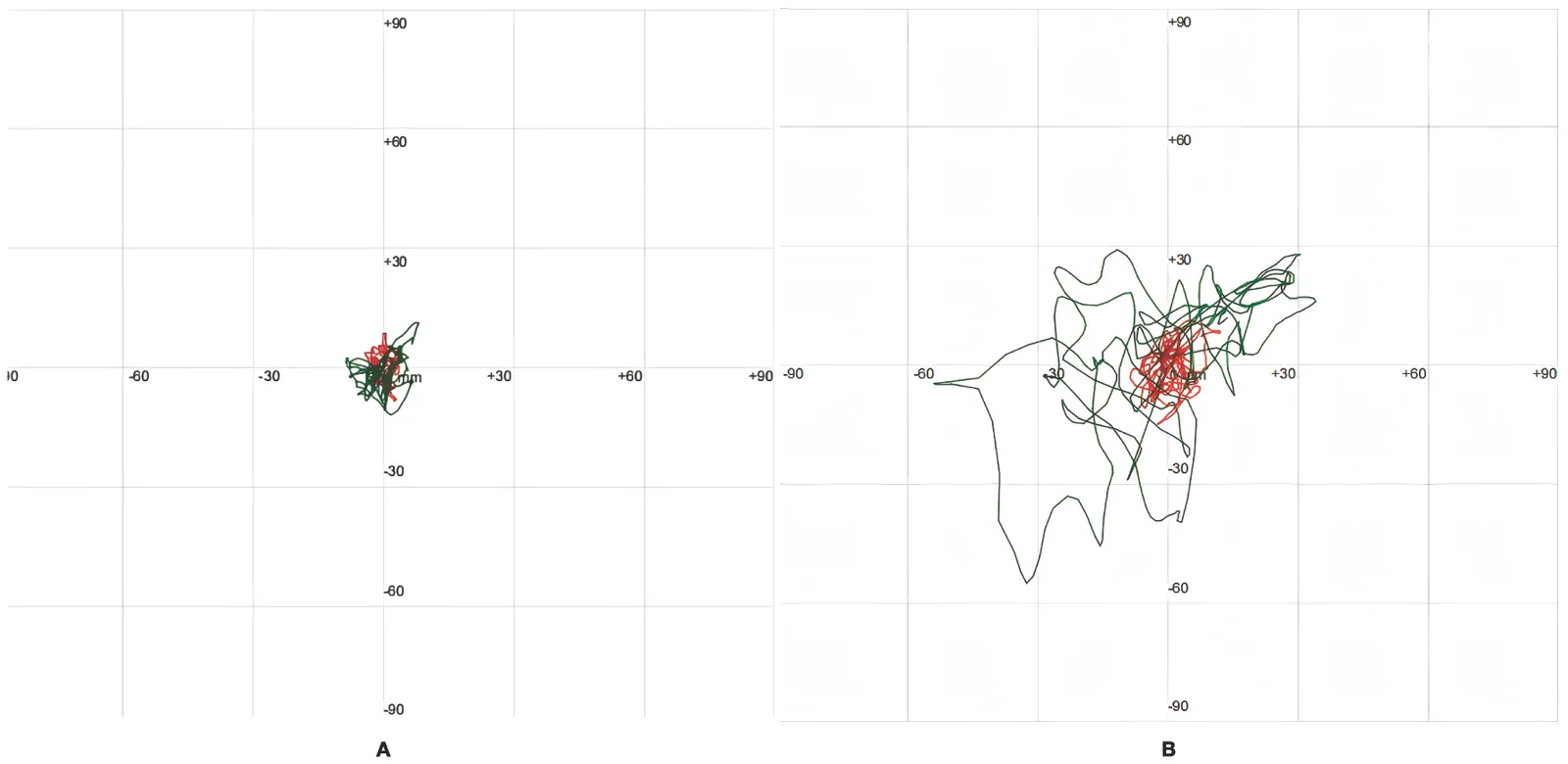
Representative trajectories of the patient’s center of pressure. **(A)** indicates a firm surface; **(B)** indicates a soft surface; Within each panel, the green line indicates the trajectory with eyes closed, and the red line indicates the trajectory with eyes open.

Participants assumed a bipedal stance with feet symmetrically positioned relative to the central axis (A1-A5). The feet were adducted (heel-to-toe), with the posterior calcanei aligned along a transverse axis. The second toes were oriented toward coordinates A8 (left) and A2 (right), positioning the intermalleolar line approximately 3 cm distal to the transverse axis A3-A7 (**Figure 1**). Participants maintained a forward gaze focused on a fixed point on the wall at eye level. Four distinct testing conditions were evaluated sequentially:

1. T1 (Firm Surface, Eyes Open [FI-EO]): This baseline condition required integration of visual, proprioceptive, and vestibular inputs.
2. T2 (Firm Surface, Eyes Closed [FI-EC]): In this condition, somatosensory and vestibular systems served as the primary sensory modalities for balance control.
3. T3 (Foam Surface, Eyes Open [FO-EO]): Sensory reliance shifted predominantly to vision and the vestibular system due to altered proprioceptive input from the compliant surface.
4. T4 (Foam Surface, Eyes Closed [FO-EC]): Representing the most challenging condition, this protocol eliminated reliable visual and proprioceptive cues, thereby isolating and assessing the vestibulospinal contribution to postural stability.

Each condition was maintained for 30 seconds and repeated twice; mean values were calculated for analysis. Participants were strictly instructed to avoid stepping, head movements, or arm motions throughout the trials. Clinicians refrained from engaging in conversation with participants during data acquisition to prevent auditory cueing.

#### 2.2.2 Static balance function index

X-axis center of pressure (X-COP): Mediolateral COP trajectory. The COP refers to the mean displacement of the center of pressure. A larger absolute value of the COP indicates poorer balance stability. The normal range is within 15 mm.

Y-axis center of pressure (Y-COP): Anteroposterior COP Trajectory. The normal range is within 15 mm.

Center of Pressure (COP) Envelope Area (COP-EA) :The postural sway area, defined as the area enclosed by the trajectory of the body’s COP and reflecting the overall amplitude of postural sway, is a key metric in balance assessment, where a smaller area generally indicates better postural stability; the established normal reference range for this area is typically below 200 mm^2^, while a value exceeding 400 mm^2^ is considered clinically abnormal, suggesting significantly impaired balance control.

Center of Pressure Path Length (COP-PL): The total trajectory length of the COP reflects proprioceptive postural control and spinal reflexive regulation of posture. A shorter trajectory length per unit area generally indicates superior motor control capability (12). The normal reference range for this parameter is typically within 250 mm, while a value exceeding 1000 mm suggests significant impairment.

Romberg ratio: The Romberg quotient is defined as the ratio of the area (or total length) of the center-of-pressure trajectory during quiet standing with eyes closed to that with eyes open. This measure quantifies the degree of dependence on proprioception for postural control by effectively eliminating visual input, thereby aiding in the assessment of potential impairment in the proprioceptive and vestibular systems. A normal Romberg quotient value is greater than 1.However, a value significantly exceeding 1 indicates an over-reliance on visual information for maintaining balance, suggesting impaired somatic proprioception (12). The postural stability was evaluated using two distinct Romberg indices: the Romberg ratio based on COP Trajectory Length (RRT), which quantifies the amount of postural sway, and the Romberg ratio based on COP Envelope Area (RRA), which assesses the spatial extent of center-of-pressure oscillations.

### 2.3 Statistical analyses, statistical methods

Statistical analyses were performed using SPSS version 26. 0 and R version 4. 3. 3. Continuous variables were assessed for normality using the Shapiro–Wilk test. Normally distributed variables are presented as mean ± standard deviation (x̄ ± s) and compared between groups using independent-sample ttests; non-normally distributed variables are expressed as median (interquartile range) [M (IQR)] and compared using the Mann–Whitney Utest. Categorical variables are described as frequencies (percentages), and between-group comparisons were performed using the χ^2^ test. Effect sizes are reported as Cohen’s d with its 95% confidence interval.

Sample size consideration: This was an exploratory cross-sectional study with a fixed consecutively enrolled sample. A *post hoc* power analysis was conducted for the primary exposure–outcome association (center-of-pressure path length under the T3 condition) using G Power version 3. 1.9. 7. For the smallest observed effect size in the study (T1 COP-PL, Cohen’s d= 0.40), the achieved power was approximately 0.46, indicating that interpretations for this particular metric should be made cautiously. In contrast, for the primary between-group difference metric (T3 COP-PL, Cohen’s d=1.45), the power exceeded 0.99, suggesting that the existing sample was adequate to reliably detect larger between-group functional differences. Nomogram development followed the events-per-variable (EPV) principle: the initial LASSO candidate pool comprised 7 predictors (demographic and balance parameters), and the final nomogram retained 4 non-zero coefficients (sex, T1 COP-PL, T3 COP-PL, and T4/T3 RRA). With 97 prediabetes events, the EPV was 24. 3 (>10), supporting the stability of logistic regression estimation. Internal validation was further confirmed via 1,000 bootstrap resamples. Owing to the single-center sample size, the generalizability of the model still warrants confirmation in external multicenter cohorts.

Model construction and validation: With prediabetes as the outcome, sex was pre-specified as a clinical covariate. LASSO regression with 10-fold cross-validation (minimum λ) was applied to the 7 candidate predictors; the resulting non-zero variables together with sex were entered into a multivariable logistic regression model (all variance inflation factors<5) to construct the nomogram. Discriminability was evaluated by the area under the receiver operating characteristic curve (AUC). Calibration was assessed using the Hosmer–Lemeshow test, calibration plot, and Brier score. Internal validation was performed via 1,000 bootstrap resamples (encompassing the full pipeline of LASSO variable selection and logistic modeling) to derive optimism-corrected C-statistics, calibration slope, and intercept. Two-sided *P* < 0.05 was considered statistically significant.

## 3 Results

### 3.1 Demographics and disease characteristics

A total of 97 patients and 44 healthy individuals were enrolled in this study. The analysis showed that the mean age of the Pre-DM group was 56. 290 years, with 31 males (31.959%). In the healthy control group, the mean age was 54. 640 years, with 17 males (38. 636%). No statistically significant differences were observed between the two groups in terms of sex, age, or BMI (*P*>0.05). All glycemic parameters were significantly higher in the prediabetes group than in the normoglycemic control group (all *P* < 0.001). Details are provided in **Table 1**.

**Table 1:** Clinical features and biochemical parameters of patients. [M(IQR)/x̄ ± s].

Variables	Pre-DM group	Healthy controls group	χ^2^/t/Z	*P*
Male/Female	31/66	17/27	0.601	0.438
Age (yrs)	57.000(14.500)	57.000(20.750)	0.312	0.755
BMI	22.993(3.500)	24.233(3.940)	-1.953	0.051
FBG (mmol/L)	6.01 (5.31, 6.75)	4.52 ± 0.38	-8.482	<0.001
2hPG (mmol/L)	7.89 (6.17, 10.08)	6.16 ± 0.55	-4.979	<0.001
HbA1c(%)	6.40 (6.00, 7.00)	4.75 ± 0.36	-9.448	<0.001

BMI, body mass index; FBG, fasting blood glucose; 2hPG, 2-hour postprandial glucose; HbA1c, glycated hemoglobin A1c.

### 3.2 Comparison of static balance indicators between groups

Static balance indicators were compared between the Pre-DM group and the healthy control group. Under the T1 condition, no significant differences were observed in the X-COP or the COP-EA between the two groups (*P*>0.05). However, the Y-COP and COP-PL showed statistically significant differences (*P*<0.05). Under the T2 condition, none of the static balance indicators exhibited significant differences between the groups ((*P*>0.05). In contrast, the RRA and RRT differed significantly between the Pre-DM and control groups (*P*<0.05). Detailed data are summarized in **Table 2-1**.

**Table 2-1:** Comparison of static postural stability parameters between the two groups while standing on a firm platform.

Item	Pre-DM group	Healthy controls group	t/Z	*P*
T1				
X-COP	-2.000(9.500)	-1.534 ± 4.708	0.045	0.964
Y-COP	0.000(24.500)	4.500(5.750)	-2.101	0.036
COP-EA	188.500(166.500)	254.318 ± 110.093	-1.273	0.203
COP-PL	303.323 ± 74.922	342.273 ± 115.329	2.052	0.044
T2				
X-COP	-2.000(7.630)	-1.375 ± 4.720	-0.060	0.952
Y-COP	6.250(5.250)	4.500(21.500)	-0.810	0.418
COP-EA	336.000(277.250)	296.500(226.380)	1.595	0.111
COP-PL	391.000(141.630)	406.800 ± 110.622	1.609	0.108
T2/T1				
RRA(%)	168.627(141.800)	142.964(84.790)	2.754	0.006
RRT(%)	148.998(45.010)	127.755(56.630)	3.275	0.001

X-COP, X-axis center of pressure; Y-COP, Y-axis center of pressure; COP-EA, Center of Pressure Envelope Area; COP-PL, Center of Pressure Path Length; RRA, Romberg ratios based on sway area; RRT, Romberg rate based on trajectory length.

Compared to the control group, significant differences(*P*<0.05) were observed in the Pre-DM group under conditions T3 and T4 for the parameters of Y-COP、COP-EA, and COP-PL. However, no significant difference was found in the X-COP (*P*>0.05). Furthermore, both the RRA and RRT derived from the T4/T3 conditions showed statistically significant differences between the Pre-DM group and the control group (*P*<0.05). For details, see **Table 2-2**.

**Table 2-2:** Comparison of static postural balance parameters between the two groups during quiet standing on a foam surface.

Item	Pre-DM group	Healthy controls group	t/Z	*P*
T3				
X-COP	-4.500(11.250)	-2.750(7.750)	-0.799	0.424
Y-COP	8.691 ± 20.474	2.727 ± 14.508	2.360	0.020
COP-EA	498.000(318.250)	244.250(206.750)	6.463	0.000
COP-PL	590.500(187.750)	353.000(129.750)	7.084	0.000
T4				
X-COP	-4.629 ± 8.270	-2.000(6.380)	-1.534	0.125
Y-COP	12.680 ± 21.020	4.500(16.130)	2.379	0.017
COP-EA	2472.000(1939.500)	551.750(545.380)	9.015	0.000
COP-PL	1363.000(627.250)	558.000(475.250)	8.208	0.000
T4/T3				
RRA(%)	494.400(406.050)	198.589(98.600)	7.654	0.000
RRT(%)	241.596(91.960)	150.913(70.820)	6.532	0.000

X-COP, X-axis center of pressure; Y-COP, Y-axis center of pressure; COP-EA, Center of Pressure Envelope Area; COP-PL, Center of Pressure Path Length; RRA, Romberg ratios based on sway area; RRT, Romberg rate based on trajectory length.

### 3.3 LASSO regression analysis of static balance function

**Figure 2:**
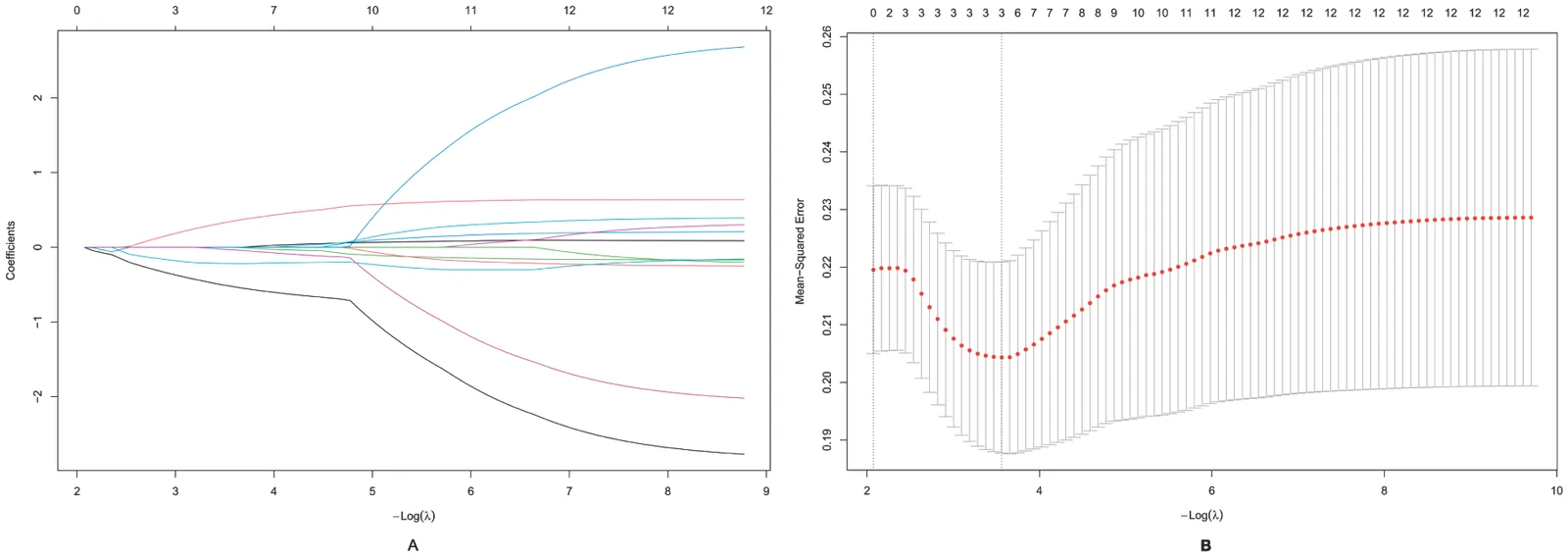
Candidate variables associated with prediabetes status selected by LASSO regression. **(A)** Coefficient curves of 12 observed indicators; **(B)** LASSO regression with 10 fold cross validation to select the best observation indicators.

Using prediabetes status (yes/no) as the dependent variable, we performed LASSO regression for dimensionality reduction and variable selection, incorporating covariates that differed significantly between groups in **Table 2**. The optimal penalty parameter (λ) was identified via 10-fold cross-validation by minimizing the cross-validation error (**Figure 2**). Four variables with non-zero coefficients were ultimately retained: T1 COP-PL, T3 COP-PL, T4/T3 RRA, and T3 RRA. These findings suggest that specific static postural balance parameters are associated with Pre-DM.

### 3.4 Multivariable logistic regression analysis of static balance function

Using Pre-DM status as the dependent variable, we incorporated the four variables selected by LASSO regression—T1 COP-PL, T3 COP-PL, T4/T3 RRA, and T4/T3 RRT—alongside prespecified clinical covariates (sex, age, BMI) into a multivariable logistic regression model. All variables included exhibited variance inflation factors (VIF) below 5, indicating no severe multicollinearity (**Table 3-1**).

**Table 3-1:** Collinearity analysis of variables.

Item	B	SE	β	*P*	VIF
T1 COP-PL	-0.001	0.000	-0.183	0.004	1.103
T3 COP-PL	0.001	0.000	0.514	<0.001	1.063
T4/T3 RRA	0.000	0.000	0.230	0.029	3.038
T4/T3 RRT	0.001	0.001	0.189	0.071	3.040
Sex	0.095	0.062	0.097	0.125	1.116
Age	-0.001	0.003	-0.013	0.839	1.134
BMI	0.002	0.009	0.016	0.810	1.170

COP-PL, Center of Pressure Path Length; RRA, Romberg ratios based on sway area; RRT, Romberg rate based on trajectory length; VIF, Variance Inflation Factor. BMI: Body Mass Index. B, Unstandardized Coefficient; β, Standardized Coefficient; SE, Standard Error; OR, Odds Ratio; Wald, Wald Test.

Subsequently, forward stepwise selection with the likelihood ratio criterion was performed, retaining sex, age, BMI,T1 COP-PL,T3 COP-PL,T4/T3 RRA, and T4/T3 RRT (all VIF<5; **Table 3-2**). Multivariable logistic regression analysis revealed that female sex (vs. male; OR=0.208, 95% CI:0.044–0.973; *P* = 0.046), T1 COP-PL (OR = 0.990, 95%CI: 0.980–0.999; *P* = 0.034), T3 COP-PL (OR = 1.016, 95% CI:1.008–1.023; *P* < 0.001), and T4/T3 RRA (OR = 1.013, 95%CI: 1.007–1.020; *P* < 0.001) were independently associated with prediabetes status.

**Table 3-2:** Multiple factor regression analysis of two groups static balance function index.

Item	β	SE	Wald	*P*	OR	95%CI
Sex (women)	1.571	0.788	3.979	0.046	0.208	0.044~0.973
T1 COP-PL	-0.010	0.005	4.470	0.034	0.990	0.980~0.999
T3 COP-PL	0.016	0.004	16.705	<0.001	1.016	1.008~1.023
T4/T3 RRA	0.013	0.003	17.018	<0.001	1.013	1.007~1.020

COP-PL, Center of Pressure Path Length; RRA, Romberg ratios based on sway area; β: Standardized Coefficient; SE: Standard Error; OR, Odds Ratio; Wald, Wald Test; 95%CI, 95%CI, 95% Confidence Interval.

Upon further inclusion of FBG, 2hPG, and HbA1c, the logistic model exhibited complete separation (OR for HbA1c=1.33×10^6^, 95%CI: 125.79–1.41×10^10^). This numerical instability reinforces our primary modeling strategy of adjusting solely for age, sex, and BMI.

### 3.5 Nomogram development and assessment of predictive strength​

**Figure 3:**
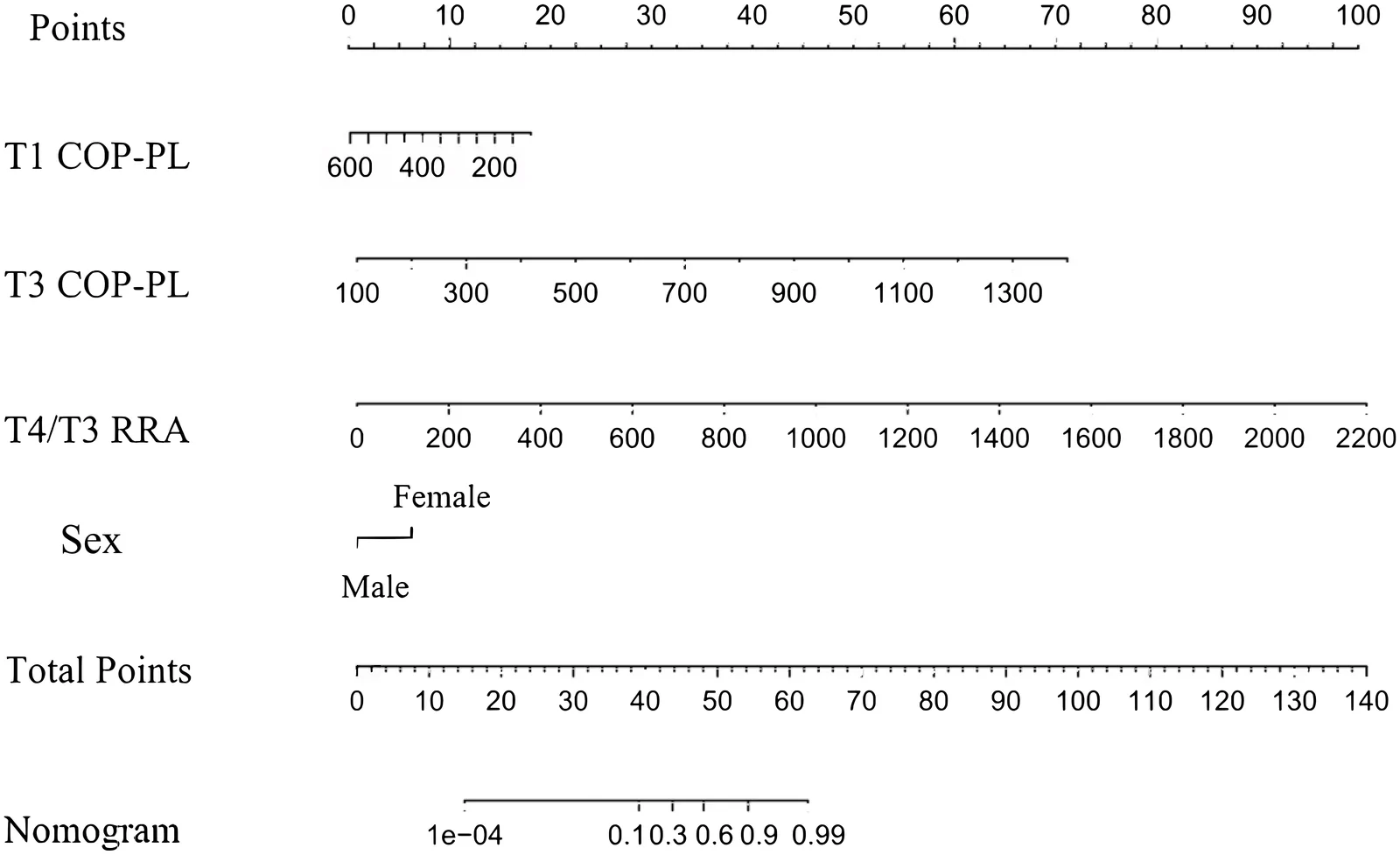
Nomogram model for factors associated with prediabetes status.

Building on the independent predictors identified via multivariable logistic regression, a nomogram was constructed using R version 4.4.1 with the rmspackage (**Figure 3**). The predictive model incorporating these variables demonstrated excellent discrimination, with an AUC of 0.972 (95% CI: 0.950-0.995; *P* < 0.001). At the optimal cutoff, the model yielded a sensitivity of 0.701 and a specificity of 1.000.

**Figure 4:**
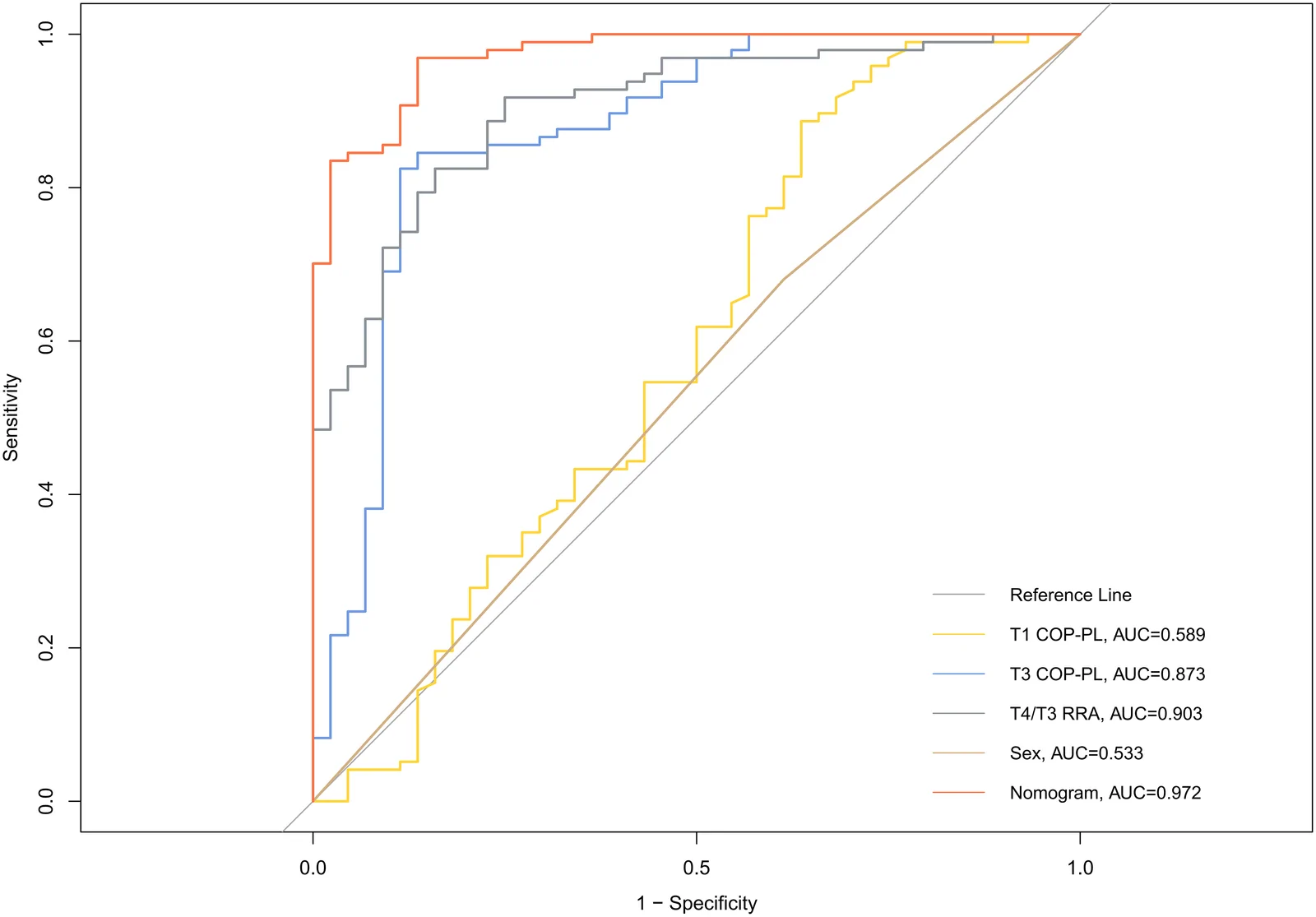
ROC curves of the nomogram and individual variables for discriminating prediabetes from normoglycemic controls.

The AUC for T1 COP-PL was 0.411 (< 0.5), and the corrected AUC after label reversal was 0.589. The predictive utility of individual variables for Pre-DM, as assessed by ROC analysis, showed AUC values of 0.533 for sex, 0.589 for COP-PL at T1, 0.873 for COP-PL at T3, and 0.903 for the T4/T3 RRA ratio. The corresponding sensitivity and specificity for these variables were 0.680 and 0.386, 0.887 and 0.364, 0.825 and 0.886, and 0.918 and 0.750, respectively. Notably, the multivariable logistic regression model combining all four factors exhibited superior performance, achieving an AUC of 0.972 (95% CI: 0.950 to 0.995; *P* < 0.001). This integrated model yielded a sensitivity of 0.701 and a specificity of 1.000, demonstrating outstanding discriminatory ability (see **Table 4**; **Figure 4**).

**Table 4:** Area under curve, 95% confidence interval.

Indicator	AUC	95% CI	*P*	Sensitivity	Specificity	Youden’s index
Sex	0.533	0.430~0.637	0.526	0.680	0.386	0.066
T1 COP-PL	0.589	0.478~0.700	0.090	0.887	0.364	0.251
T3 COP-PL	0.873	0.803~0.943	<0.001	0.825	0.886	0.711
T4/T3 RRA	0.903	0.852~0.954	<0.001	0.918	0.750	0.688
Logistic Regression Model	0.972	0.950~0.995	<0.001	0.701	1.000	0.701

COP-PL, Center of Pressure Path Length; RRA, Romberg ratios based on sway area.

### 3.6 Hosmer-Lemeshow calibration curve for validating the nomogram association model

**Figure 5:**
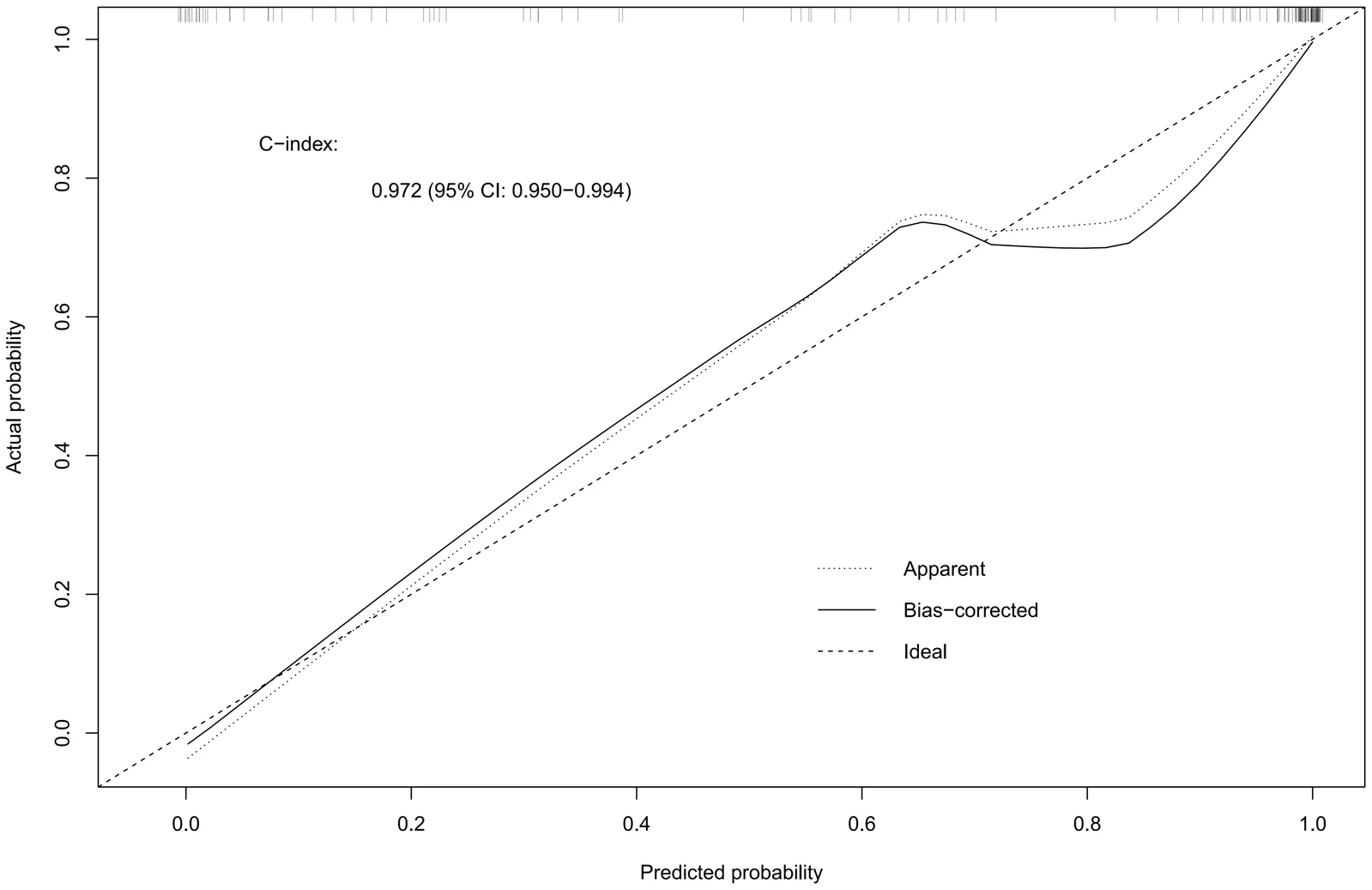
Hosmer-Lemeshow calibration plot of the nomogram model.

Evaluation of calibration, performed via the Hosmer-Lemeshow test and calibration curves, revealed favorable performance characteristics for the constructed nomogram. The model exhibited superior discriminative power, evidenced by a C-index of 0.972 (95% CI: 0.950 to 0.994). Assessment of calibration indicated a close alignment of the “Apparent” curve with the diagonal ideal line, reflecting a good fit in the training set. Notably, the “Bias-corrected” curve was observed to be nearly congruent with the ideal line, indicating minimal overfitting and robust internal validation. It is concluded that the model demonstrates satisfactory calibration, with a high level of agreement between the model’s predicted probabilities and the actual observed event rates (see **Figure 5**).

### 3.7 Internal validation with bootstrap resampling

**Figure 6:**
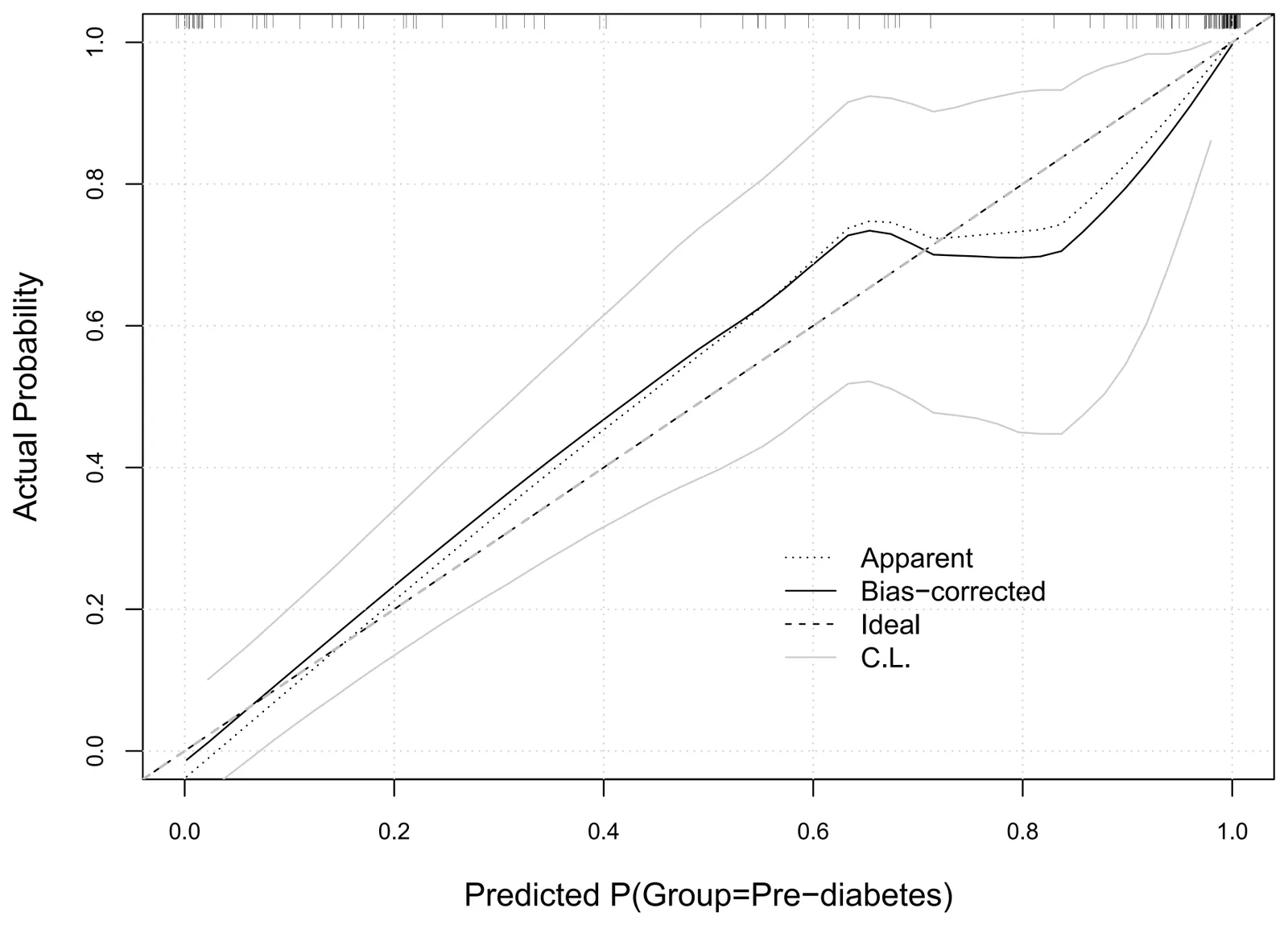
Bootstrap internal validation calibration plot of the nomogram model.

The nomogram was internally validated using 1000 bootstrap resamples. The bias-corrected AUC was 0.964 (95% CI, 0.941-0.991), indicating excellent discrimination. The calibration slope was 0.806, the calibration intercept was −0.011, and the Brier score was 0.075, reflecting close agreement between predicted probabilities and observed outcomes and confirming good overall calibration (see **Figure 6**).

### 3.8 Calculation of effect sizes for associated factors

The effect sizes for COP-PL under Condition T1, COP-PL under Condition T3, and the T4/T3 RRA ratio were quantified using Cohen’s d. According to conventional thresholds (Cohen, 1988), where |d|≥0.2 represents a small effect, |d|≥0.5 a medium effect, and |d|≥0.8 a large effect, the calculated values were as follows: Cohen’s d= -0.40 (95% CI: -0.73 to -0.06) for COP-PL (T1), indicating a small to medium effect; d=1.45 (95% CI: 1.08 to 1.81) for COP-PL (T3); and d= 1.39 (95% CI: 1.03 to 1.75) for the T4/T3 RRA ratio. The effects for the latter two variables are considered large, suggesting substantial differences in static balance function associated with these parameters in the context of Pre-DM. The extent of static balance dysfunction in Pre-DM patients was markedly influenced by the complexity of the postural task. A significant and clinically relevant decline, supported by large effect sizes, was specifically evident under the challenging sensory conditions of T3 and T4/T3, whereas performance in the simpler T1 condition showed negligible differences.

## 4 Discussion

The maintenance of human body balance requires the central nervous system to accurately integrate information from the relevant sensory systems, namely vision, proprioception, and the vestibular system (collectively known as the sensory triad for balance). Dysfunction in any one of these systems can compromise postural stability (13). A dysfunction in any one of these three systems can compromise overall postural stability. Research indicates that each component contributes distinct sensory inputs: proprioception primarily conveys signals related to force and movement from the feet and the support surface; the vestibular system transmits information regarding the position and movement of the head in response to gravity, whether active or passive; and the visual system provides information on the motion of the head relative to the surrounding environment (14). Static balance impairment is primarily associated with a decline in the integrative capacity of the balance triad (vestibular, visual, and proprioceptive systems). Patients with Pre-DM exhibit increased reliance on visual input when sensory conditions are compromised, such as under visual deprivation (15). Studies have confirmed that diabetic microangiopathy and peripheral neuropathy serve as the pathophysiological basis for balance dysfunction in these patients (16). Evidence indicates that diabetic neuropathy also exists in patients with Pre-DM. A statistically significant association has been established between Pre-DM and the occurrence of peripheral neuropathy. The prevalence of peripheral neuropathy in the Pre-DM population is ≥10%, which is higher than that in normoglycemic individuals. This association appears to be more pronounced in individuals with IGT (17–21). Furthermore, evidence suggests that individuals with Pre-DM may already exhibit early signs of microvascular complications, including, but not limited to, retinopathy, nephropathy, and neuropathy (22–24). Early detection and lifestyle modification are essential for halting the progression of neurological damage and improving quality of life. Nevertheless, early signs of neuropathy and microvascular complications in Pre-DM patients are frequently overlooked, highlighting an urgent need for greater clinical vigilance and the implementation of routine screening.

### 4.1 Comparative analysis of static balance parameters between groups

No statistically significant difference was observed in the X-COP between the Pre-DM and control groups. The X-COP parameter reflects mediolateral sway (coronal plane) of the body’s center of pressure. This finding suggests that postural stability in the coronal plane remains relatively preserved at this stage of prediabetes.

In contrast, significant between-group differences were identified for the Y-COP—a measure of anteroposterior sway (sagittal plane)—specifically under conditions T1, T3, and T4. A Y-COP absolute value exceeding 15 mm is indicative of reduced sagittal plane stability, potentially arising from impaired proprioceptive input or compromised central integration, and is associated with an elevated risk of falls. The observed alterations in Y-COP under conditions that manipulate visual and/or somatosensory input suggest a close relationship with functional changes in the visual and vestibular systems. Consequently, these results imply that rehabilitation strategies for individuals with Pre-DM might benefit from incorporating targeted vestibular training and exercises aimed at enhancing sagittal plane postural control.

COP-PL quantifies the total activity of postural adjustments, where an increased length reflects reduced efficiency in postural control. A COP-PL value exceeding 250 mm suggests impairment in proprioceptive and sensorimotor integration, while a value greater than 1000 mm indicates significant abnormalities in balance control. Under T1, T3, and T4 conditions, the COP-PL values in the Pre-DM group demonstrated statistically significant differences compared to the control group, highlighting its association with alterations in visual and vestibular function. COP-EA represents the spatial extent of center-of-pressure sway. An enlarged area indicates increased postural sway amplitude, potentially resulting from failed integration of visual and proprioceptive inputs. A COP-EA value greater than 200 mm^2^ signifies insufficient overall static balance stability, whereas a value exceeding 400 mm^2^ suggests a high-risk state. Under T3 and T4 conditions, the Pre-DM group exhibited significantly larger Y-COP displacements compared to the control group, further supporting its close relationship with functional changes in the visual and vestibular systems.

These findings align with previous research demonstrating correlations between FIns, HOMA-IR, and static balance function, where both HOMA-IR and FIns show negative correlations with vestibular and visual function (10). This suggests that individuals with Pre-DM exhibit deficits in visual and vestibular systems, compromising their ability to maintain static postural stability. Substantial evidence indicates that balance impairments in patients with established DM are characterized by a significant decline in vestibular function, coupled with an increased reliance on visual cues for postural control (6, 14). This is primarily attributed to diabetic retinopathy (DR), a common complication of T2D that contributes to gait instability and increased fall risk (25). Animal and clinical studies further confirm that diabetes adversely affects vestibular function, and this vestibular dysfunction is directly associated with impaired balance and a higher incidence of falls (25). Within the Pre-DM population, the prevalence of DR is estimated to be between 6% and 8%, with early manifestations primarily involving neurodegenerative changes and initial microvascular damage (26, 27).

Therefore, during the Pre-DM stage, patients already exhibit significant impairment in static balance function. This deficit becomes particularly pronounced under challenging conditions, such as visual deprivation or when standing on unstable, compliant support surfaces. These conditions exacerbate the underlying abnormalities in visual, proprioceptive, and vestibular input, and highlight the diminished capacity for effective central sensory integration, leading to markedly impaired postural stability.

### 4.2 Characteristics of Romberg rate variations

The RRT is defined as the ratio of the center of pressure path length with eyes closed to that with eyes open during upright stance; a longer path indicates poorer balance control. Under the T2/T1 and T4/T3 conditions, the RRT values in the Pre-DM group were significantly higher than those in the control group (ratio >1), suggesting that these patients exhibited increased visual dependency accompanied by impaired somatosensory proprioception. This aligns with prior research indicating that poor glycemic control is associated with proprioceptive deficits in type 2 diabetes, where worse glycemic management correlates with more pronounced proprioceptive dysfunction (28).

The RRA represents the ratio of the sway area with eyes closed to that with eyes open, reflecting the spatial magnitude of postural sway: a larger area denotes poorer postural control or coordination. Similarly, under the T2/T1 and T4/T3 conditions, the RRA was markedly elevated in the Pre-DM group compared to controls (ratio >1), indicating an overreliance on visual cues to compensate for deficient proprioceptive feedback. These findings imply abnormalities in both proprioceptive and vestibular function in Pre-DM, particularly a decline in the reliability of proprioception, leading to greater dependence on vision. Agrawal Y. et al. have proposed that Romberg testing can help identify vestibular dysfunction, noting that adults with diabetes have a 70% higher likelihood of vestibular impairment compared to non-diabetic individuals (29).

Notably, the RRT and RRA values under the T4/T3 condition were substantially higher than those under T2/T1.This suggests that when proprioceptive input is disrupted by the foam surface and visual input is simultaneously removed (eyes closed), individuals with Pre-DM demonstrate an impaired ability to effectively integrate and utilize remaining sensory information to maintain balance.

### 4.3 Factors associated with prediabetes status and nomogram model analysis

Evidence suggests that individuals with Pre-DM and T2DM often exhibit impairments in static balance function (16). This study aimed to investigate the specific relationships between Pre-DM and quantifiable static balance parameters. Our results demonstrate significant associations between Pre-DM status and several balance metrics, including COP-PL under condition T1, COP-PL under condition T3, as well as the RRA and the RRT under the T4/T3 condition. These results suggest that early manifestations of subclinical neuropathy or central integration deficits may occur prior to blood glucose levels fully meeting the diagnostic criteria for DM, which aligns with the static balance dysfunction observed in our Pre-DM cohort (30). Integrating LASSO feature selection with subsequent multivariable logistic regression, we identified T1 COP-PL, T3 COP-PL, and T4/T3 RRA as independent correlates of prediabetes status. Crucially, these three postural stability metrics remained retained in the final adjusted model (**Table 3-2**) after accounting for age, sex, and BMI. These findings suggest that the observed deficits in vestibulo-proprioceptive integration and reduced postural control efficiency—as indexed by these parameters—are not merely secondary to chronological aging or anthropometric variability, but rather implicate early glycemic dysregulation per se.

Among the identified predictors, T4/T3 RRA exhibited the largest standardized coefficient (β=0.013) with an OR of 1.013 (95% CI:1.007–1.020). This indicates that a greater increase in postural sway under concurrent visual and proprioceptive perturbation (relative to proprioceptive disruption alone) correlates positively with the probability of prediabetes. This observation aligns with the systematic review by Jiang X et al., which reported that subclinical peripheral nerve dysfunction may manifest as early as the prediabetic stage, primarily presenting as significantly increased anteroposterior center-of-pressure excursion during eye-closed stance compared to healthy controls (31). Our study extends this finding by applying the Romberg ratio (T4/T3) to quantify the gradient of postural control efficiency across varying sensory conditions, thereby providing richer phenotypic information than single-condition sway metrics.

The second strongest predictor was T3 COP-PL (OR = 1.016, 95% CI:1.008–1.023). Under the T3 condition (foam surface with eyes open), individuals must rely predominantly on visual and vestibular inputs to maintain upright stance following proprioceptive deprivation. The elevated COP-PL in this condition likely reflects early impairments in multisensory integration among prediabetic individuals. Supporting this notion, Grewal et al. demonstrated that patients with diabetic peripheral neuropathy (DPN) exhibited significant improvements in postural stability following tailored sensor-based motor training (32), underscoring the presence of sensorimotor integration deficits distinct from healthy populations.

Conversely, T1 COP-PL showed a negative association with prediabetes risk (OR = 0.990, 95% CI:0.980–0.999). This inverse directionality suggests that under the least challenging condition—standing on a firm surface with eyes open—individuals with prediabetes do not exhibit exaggerated sway; rather, a subtle reduction in sway path length emerged in the multivariable context. However, caution is warranted in interpreting this finding. The univariate effect size for T1 COP-PL was modest (Cohen’s d=–0.40), and its standalone discriminatory ability was limited (AUC<0.70). Consequently, this metric should not be considered a robust standalone screening tool for prediabetes.

Furthermore, female sex was assigned a higher predicted probability in the nomogram (reference group: females; OR= 0.208, 95% CI: 0.044–0.973). This implies that males carried a nearly fivefold higher odds of having prediabetes compared to females within this cohort. Such a sex disparity may be partially attributed to menopausal transitions and associated hormonal fluctuations, which have been linked to accelerated declines in muscle mass (sarcopenia) and earlier-onset deficits in postural stability among women with metabolic disturbances (33, 34).

Although regression models are essential for analyzing relationships among complex clinical variables, their results are typically presented as mathematical equations and consequently lack intuitive interpretability. In contrast, nomogram models enable quantitative scoring of complex clinical variables, allowing individualized prediction probabilities to be calculated intuitively and displayed through a graphical interface (35). To further investigate the association between static balance indices and prediabetes, a nomogram was constructed based on multivariable logistic regression results, providing a visual representation of the relative contribution of each factor. The nomogram revealed that the RRA under the T4/T3 condition carried the greatest weight, followed by COP-PL under the T1 and T3 conditions and sex. Notably, the RRA exhibited a broad point span, indicating that its association with prediabetes was the most prominent. Receiver operating characteristic curve analysis demonstrated that the model combining these four variables had good discriminatory ability in differentiating prediabetes from normoglycemia, with an area under the curve of 0.972 (95% CI: 0.95–0.995), a sensitivity of 0.701, and a specificity of 1.0. These findings suggest that the model can effectively distinguish between the two groups within the current study sample; however, its generalizability still requires validation in external independent samples.

Model calibration was evaluated using the Hosmer–Lemeshow test and a calibration curve, which demonstrated strong agreement between the probabilities predicted by the nomogram and the observed probabilities. In terms of discrimination, the AUC for the model combining the four variables was higher than that of any single indicator. The combined model exhibited the best discriminatory performance, followed by the RRA under the T4/T3 condition and the COP-PL under the T3 condition. To further assess model stability, internal validation was conducted using 1,000 bootstrap resamples. The optimism-corrected AUC was 0.964 (95% CI, 0.941–0.991), indicating that the model retained good ability to differentiate prediabetes from normoglycemia in this sample. The calibration intercept was −0.011, close to zero, suggesting no systematic overestimation or underestimation of predicted probabilities. The Brier score was 0.075, well below 0.25, further confirming close agreement between the predicted probabilities and the observed outcomes. The calibration slope was 0.806, slightly lower than the ideal value of 1, which may reflect mild overfitting; consequently, predicted effects should be appropriately shrunk in practical applications. Nevertheless, this slope remains within an acceptable range, and the low Brier score indicates that this deviation has a limited impact on the interpretation of the results.

In this study, Cohen’s d values were calculated for T1 COP-PL, T3 COP-PL, and T4/T3 RRA, yielding −0.40, 1.45, and 1.39, respectively, all corresponding to medium-to-large effect sizes. These results indicate that the associations between these static balance parameters and prediabetes were of substantial magnitude in the current sample.

In summary, this set of candidate functional indicators—T1 COP-PL, T3 COP-PL, and T4/T3 RRA—collectively delineates the overall performance of static balance, while each may point to distinct pathophysiological aspects such as sensory reweighting capacity and postural control efficiency. The exploratory value of this study lies not in replacing established biochemical tests such as blood glucose and glycated hemoglobin, but in offering supplementary information from a functional perspective for the comprehensive assessment of individuals with prediabetes. The potential implication is that these indicators may reveal changes in static balance function that conventional biochemical markers cannot directly capture, thereby adding an additional observational dimension to the existing evaluation framework.

## 5 Conclusion

The primary value of this study lies in the exploratory identification of clinical differences and associations between altered static balance function and prediabetes, as well as in the initial screening of a candidate set of functional indicators—T1 COP-PL, T3 COP-PL, T4/T3 RRA, and sex—and the attempt to construct a nomogram-based association model. This model may be regarded as a proof-of-concept; its clinical relevance and generalizability remain to be examined and refined in subsequent studies.

Several limitations should be acknowledged. This was a single-center, clinical observational study with a modest sample size, and potential confounders such as physical activity and neuropathy were not comprehensively measured or controlled. Longitudinal follow-up data on the progression from prediabetes to diabetes were not available, precluding assessment of the temporal changes or predictive value of these indices. Moreover, the model has not been validated in an independent external cohort, and its broader applicability awaits further confirmation. To address these issues, our group plans to conduct a multicenter, prospective study with more comprehensive and precise measurement of confounders; to implement long-term follow-up of a fixed cohort to evaluate the dynamic changes in static balance indices during the deterioration of glucose metabolism; and to rigorously test and optimize the nomogram model using the current data as a training set and an independent validation set.

In summary, this cross-sectional study suggests that individuals with prediabetes may exhibit declines in static balance function under complex sensory conditions, such as with eyes closed or while standing on an unstable surface. Sex, T1 COP-PL, T3 COP-PL, and T4/T3 RRA were independently associated with prediabetes status. As a candidate set of functional indicators, they may offer a supplementary observational perspective for the functional assessment of the prediabetic population; however, their clinical translational value requires further validation in larger-scale prospective studies.

## Data Availability

The data that support the findings of this study are available from the corresponding author upon reasonable request. The data are not publicly available due to privacy restrictions. Requests to access the datasets should be directed to ZW, sy2024008@fjtcm.edu.cn.

## References

[1] Tabák AG Herder C Rathmann W Brunner EJ Kivimäki M . Prediabetes: a high-risk state for diabetes development. Lancet. (2012) 379:2279–90. doi: 10.1016/S0140-6736(12)60283-9 22683128 PMC3891203

[2] He D Li J Li Y Zhu J Zhou T Xu Y . Frailty is associated with the progression of prediabetes to diabetes and elevated risks of cardiovascular disease and all-cause mortality in individuals with prediabetes and diabetes: Evidence from two prospective cohorts. Diabetes Res Clin Pract. (2022) 194:110145. doi: 10.1016/j.diabres.2022.110145 36356844

[3] Li J Xie Y Zhao MM Chen K Dong AQ Ning C . Research progress on balance function in patients with type 2 diabetes mellitus. China Modern Med. (2021) 28:42–6.

[4] Lopatin T Sakyi K Kendall B Grunberger G Haworth J . Standing balance impairment in persons with type 2 diabetes is predicted by peripheral neuropathy and vestibulopathy. Prim Care Diabetes. (2025) 19:35–9. doi: 10.1016/j.pcd.2024.10.001 39709234

[5] Jia J Meng Y Zhang LL Zhuang XM . Analysis of static balance function in patients with type 2 diabetes mellitus. J Capital Med Univ. (2019) 40:6–10.

[6] Guo S Liu ZZ Qin JW Yin LH Huang J Tao J . Characteristics of static balance function in elderly patients with type 2 diabetes mellitus under different blood pressure status. Rehabil Med. (2021) 31:112–8. 42529480

[7] Hu XX Yang XG Wang X Ma X Geng X . The influence of diabetes and age-related degeneration on body balance control during static standing: a study based on plantar center-of-pressure trajectories and principal component analysis. J Orthop Surg Res. (2023) 18:740. doi: 10.1186/s13018-023-04129-1 37775789 PMC10542244

[8] Yin L Qin J Chen Y Xie J Hong C Huang J . Impact of body mass index on static postural control in adults with and without diabetes: a cross-sectional study. Front Endocrinol (Lausanne). (2021) 12:768185. doi: 10.3389/fendo.2021.768185 35002958 PMC8739700

[9] Vargas Matamala M Tapia C Salvador Sagüez F Guerrero-Henriquez J . Postural performance assessment in aging people with diabetes and diabetic peripheral neuropathy using a Wii balance board. Disabil Rehabil. (2023) 45:1202–7. doi: 10.1080/09638288.2022.2055168 35369833

[10] Wang Z Chen Y Yang W Xie Y Li M Liang J . Static balance in prediabetic subjects: correlation study involving glucose indicators, insulin resistance and postural stability indices. Int J Gen Med. (2025) 18:5233–42. doi: 10.2147/IJGM.S547267 40949308 PMC12433247

[11] Chinese Diabetes Society . Guidelines for the prevention and treatment of type 2 diabetes in China (2020 Edition) (Part 1). Chin J Pract Intern Med. (2021) 41:668–95. doi: 10.19538/j.nk2021080106

[12] Chen Y Ou JL Chen ZM . Expert consensus on balance function assessment and clinical application of rehabilitation equipment based on pressure sensor recognition. Guangdong Med J. (2025) 46(2):181–92. doi: 10.13820/j.cnki.gdyx.20241667

[13] Liu C . Diseases of the Inner Ear. Beijing: People's Medical Publishing House (2006) p. 59–80.

[14] Huang XB Liu B . The role of the balance triad and central integration in human balance. J Audiology Speech Pathol. (2009) 17:534–6.

[15] Lu B Zhang LL Duan JT Zhuang XM Meng Y . Clinical observation of decreased static balance function in patients with impaired glucose regulation. Chin J Diabetes. (2019) 11:788 92. doi: 10.3760/cma.j.issn.1674-5809.2019.12.004 30704229

[16] Zhou Y Liu B . Mechanisms of balance dysfunction in diabetic patients. Chin J Otology. (2011) 9:365 8.

[17] Bongaerts BW Rathmann W Heier M Kowall B Herder C St ckl D . Older subjects with diabetes and prediabetes are frequently unaware of having distal sensorimotor polyneuropathy: the KORA F4 study. Diabetes Care. (2013) 36:1141 6. doi: 10.2337/dc12-0744 23275355 PMC3631873

[18] Callaghan BC Gao L Li Y Zhou X Reynolds E Banerjee M . Diabetes and obesity are the main metabolic drivers of peripheral neuropathy. Ann Clin Transl Neurol. (2018) 5:397 405. doi: 10.1002/acn3.531 29687018 PMC5899909

[19] Zeng J Xu Y Shi Y Jiang C . Inflammation role in sensory neuropathy in Chinese patients with diabetes/prediabetes. Clin Neurol Neurosurg. (2018) 166:136 40. doi: 10.1016/j.clineuro.2018.01.031 29414152

[20] Riahi R Seindareh S Aminorroaya A Ghasemi M Mehvari J Maracy MR . The relationship between prediabetes and peripheral neuropathy-a systematic review and meta-analysis. Eur J Neurol. (2025) 32:e70283. doi: 10.1111/ene.70283 40626353 PMC12235325

[21] Ziegler D Herder C Papanas N . Neuropathy in prediabetes. Diabetes Metab Res Rev. (2023) 39:e3693. doi: 10.1002/dmrr.3693 37470302

[22] Sörensen BM Houben AJ Berendschot TT Schouten JS Kroon AA van der Kallen CJ . Prediabetes and type 2 diabetes are associated with generalized microvascular dysfunction: the Maastricht Study. Circulation. (2016) 134:1339 52. doi: 10.1161/CIRCULATIONAHA.116.023446 27678264

[23] Lamprou S Koletsos N Mintziori G Anyfanti P Trakatelli C Kotsis V . Microvascular and endothelial dysfunction in prediabetes. Life (Basel). (2023) 13:644. doi: 10.3390/life13030644 36983800 PMC10057153

[24] Zhong P Liu R Zhu Z Huang W Wang W . Frailty and risk of microvascular disease in adults with prediabetes. Diabetes Metab Syndr. (2024) 18:102942. doi: 10.1016/j.dsx.2024.102942 38211481

[25] D'Silva LJ Lin J Staecker H Whitney SL Kluding PM . Impact of diabetic complications on balance and falls: contribution of the vestibular system. Phys Ther. (2016) 96:400 9. doi: 10.2522/ptj.20140604 26251477 PMC4774386

[26] Rajalakshmi R UmaSankari G Sivaprasad S Venkatesan U Kumpatla S Shanthirani CS . Prevalence and risk factors for diabetic retinopathy in prediabetes in Asian Indians. J Diabetes Complications. (2022) 36:108131. doi: 10.1016/j.jdiacomp.2022.108131 35093270

[27] Jin J Lu P . Association between prediabetes and retinopathy: a meta-analysis. Horm Metab Res. (2021) 53:801 9. doi: 10.1055/a-1678-7092 34891210

[28] Alshahrani MS Reddy RS Alshahrani A Alsubaie SF . Impact of glycemic control on shoulder proprioception in type 2 diabetes mellitus: mediating the connection-insights from a cross-sectional analysis. J Multidiscip Healthc. (2024) 17:3043 52. doi: 10.2147/JMDH.S468359 38974374 PMC11225991

[29] Agrawal Y Carey JP Della Santina CC Schubert MC Minor LB . Disorders of balance and vestibular function in US adults: data from the National Health and Nutrition Examination Survey, 2001-2004. Arch Intern Med. (2009) 169(10):938–44. doi: 10.1001/archinternmed.2009.66 19468085

[30] Huebschmann AG Scalzo RL Yang X Schmiege SJ Reusch JEB Dunn AL . Type 2 diabetes is linked to higher physiologic markers of effort during exercise. Front Clin Diabetes Healthc. (2024) 5:1346716. doi: 10.3389/fcdhc.2024.1346716 38741611 PMC11089245

[31] Jiang X Deng F Rui S Ma Y Wang M Deng B . The evaluation of gait and balance for patients with early diabetic peripheral neuropathy: a cross-sectional study. Risk Manag Healthc Policy. (2022) 15:543–52. doi: 10.2147/RMHP.S361698 35386278 PMC8977473

[32] Grewal GS Schwenk M Lee-Eng J Parvaneh S Bharara M Menzies RA . Sensor-based interactive balance training with visual joint movement feedback for improving postural stability in diabetics with peripheral neuropathy: a randomized controlled trial. Gerontology. (2015) 61:567–74. doi: 10.1159/000371846 25721132

[33] Rodrigues ST Delacosta TC Barbieri FA Paschoalino GP Gotardi GC Barela JA . Diabetic older women without peripheral neuropathy amplify body sway but are capable of improving postural stability during a saccadic gaze task. Hum Mov Sci. (2023) 92:103153. doi: 10.1016/j.humov.2023.103153 37871473

[34] Park JY Chung YJ Song JY Kil KC Lee HY Chae J . Sarcopenic obesity: a comprehensive approach for postmenopausal women. J Menopausal Med. (2024) 30:143–51. doi: 10.6118/jmm.24004 39829191 PMC11745730

[35] Cheng LN Wang MY Zhao LL Zhang L Qi C . Analysis of risk factors for elevated intraocular pressure after cataract surgery in elderly patients and construction of a nomogram model. Trans Med J. (2025) 14:61–5.

